# Trust in institutions affects vaccination campaign outcomes

**DOI:** 10.1093/trstmh/trae048

**Published:** 2024-07-30

**Authors:** David Leblang, Michael D Smith, Dennis Wesselbaum

**Affiliations:** Department of Politics and Batten School of Public Policy, University of Virginia, Charlottesville, VA 22904-4893, USA; National Oceanic and Atmospheric Administration, Economics and Social Sciences Research, Alaska Fisheries Science Center, 7600 Sand Point Way NE, Seattle, WA 98115-6349, USA; Department of Economics, University of Otago, Dunedin 9016, New Zealand

**Keywords:** COVID-19, Public Health Administration, trust, vaccination

## Abstract

**Background:**

Trust is an important driver of various outcomes, but little is known about whether trust in institutions affects actual vaccination campaign outcomes rather than only beliefs and intentions.

**Methods:**

We used nationally representative, individual-level data for 114 countries and combined them with data on vaccination policies and rates. We measured the speed of the vaccination campaign for each country using the estimated growth rate of a Gompertz curve. We then performed country-level regressions in the global sample and explored heterogeneity across World Bank development groups.

**Results:**

Globally, higher trust in institutions significantly increased vaccination rates (p<0.01) and vaccination speed (p<0.01). The effect was strong in low- and middle-income countries but statistically not significant in high-income countries.

**Conclusions:**

Our findings have implications for the design of vaccination campaigns for national governments and international organizations. The findings highlight the importance of trust in institutions when designing communication strategies around vaccination campaigns in low- and middle-income countries.

## Introduction

Trust is an important driver of various socio-economic outcomes. Studies have shown that trust increases the efficient and effective functioning of markets and economic growth.^1,2^ Research also suggests trust is a major driver of vaccine hesitancy. A study conducted in 2021, surveying 1000 individuals each in 23 countries found that almost a quarter (24.8%) acknowledged hesitancy about coronavirus disease 2019 (COVID-19) vaccines.^3^ Furthermore, trust in science has been shown to affect vaccine confidence, including confidence that vaccines are safe and effective.^4^ Research has also shown that trust in institutions affects vaccine confidence and other beliefs about vaccines; for example, that they are important or that regulators and manufacturers are competent and have good intentions.^5–8^ Earlier studies show that trust in healthcare increases willingness to obtain medical care and confidence in vaccines.^9,10^ A study sampling 33 middle- and high-income countries found that trust is positively correlated with life expectancy and negatively with mortality.^11^ Furthermore, trust in governments, science and physicians affects COVID-19 vaccine hesitancy and beliefs about vaccines generally, such as faith in their safety and efficacy.^4,5,7,12^

Well before the COVID-19 pandemic, trust in physicians and health systems was in decline and scientists were concerned about the increased scepticism about vaccines and the rapid rise of anti-vaccine movements.^13–16^ The COVID-19 pandemic accelerated these trends, culminating in the rise of various conspiracy theories.^17^ This imperils both COVID-19 vaccination efforts and other public health measures, such as lockdowns and social distancing.^18^ At the same time, trust is negatively correlated with per capita healthcare spending (ρ=−0.09, p=0.32). This non-significant correlation is likely due to a low number of observations (*N*=112). Assuming such spending increases access to prevention and treatment for COVID-19, lack of trust is preventing people from receiving the full benefits of such spending. Rates of COVID-19 cases (ρ=0.48, p=0.000) and deaths (ρ=0.35, p<0.001) are high in countries with high healthcare spending.

In this article we investigate whether trust in institutions affects vaccination rates (i.e. the share of people fully vaccinated) and the speed of the COVID-19 vaccination campaign, which we believe is an equally important outcome given the high mutation rate of the severe acute respiratory syndrome coronavirus 2 (SARS-CoV-2) virus and relates to the level of vaccine hesitancy in a country.^19^

In contrast to most articles in the related literature, we do not study beliefs or intentions about vaccinations but actual vaccination outcomes. This is important because a large amount of literature has documented that beliefs about vaccinations do not necessarily translate into getting vaccinated.^20,21^ We hypothesize trust to be a factor, if not the key factor determining the success of vaccination campaigns, because it should reduce transaction, screening and monitoring costs. Trust also allows for exchange in the absence of formal institutions and is an important driver of accessing information in networks.^22^

## Methods

### Dataset construction

We combine various datasets in this article. The key dataset is the individual-level survey data from the Gallup World Poll (GWP). Using individual-level responses in 114 countries over 8 y (2009–2017), we construct country-level averages (see Supplementary Table S.1 for a list of countries). The GWP collects information using a nationally representative sample consisting of 1000 observations for most countries (the underlying dataset contained >1 million observations). Individuals are randomly selected using the Kish grid method. In middle- and high-income countries with at least 80% telephone coverage, telephone interviews are used; face-to-face interviews are used in low-income countries. To provide reliable prevalence statistics, we pooled individual responses across all years and calculated the average for each country. Taking data from nine waves makes our measure of trust more robust to outliers and events, e.g. political scandals.^23^

### Outcome variables

The vaccination rate data were taken from the Johns Hopkins Coronavirus Research Center (4 August 2022). The vaccination rate measures the share of the population in a country that is fully vaccinated (meaning they received all prescribed doses).

We measured the vaccination speed (or vaccine hesitancy) by fitting a sigmoid function to the cumulative number of vaccines supplied to a country (per capita) over time (*t*) for each country. When COVID-19 vaccines first became available, countries sought to buy enough vaccines to cover all citizens old enough to get them. Thus vaccine supply was at first much smaller than demand. But over time it came to equal demand as production capacity grew. We are using the amount of vaccine supplied rather than the vaccination rate because we could not find appropriate high-frequency vaccination rate data by country.

We used daily data, *t*, from 12 December 2020 to 6 August 2022 (*N*=603), obtained from the Johns Hopkins Coronavirus Research Center. The vaccination speed is the non-linear least squares estimate of the growth rate parameter, β, of the Gompertz curve:^24^


(1) \begin{eqnarray*}
y = \kappa {{e}^{ - {{e}^{\alpha - \beta t}}}},
\end{eqnarray*}

where κ is the asymptote (or vaccination target) and α relates to the inflection point (α/β). We set the vaccination target to 2, implying that countries aim to achieve double vaccination for each citizen.^25^ Furthermore, only 13 countries in our sample (10.7%) have a vaccine supply:population ratio >3 and our results are robust to using κ=3. Overall, the Gompertz curve assumes a lower initial growth path, which we believe aligns with vaccination campaigns in most countries.

### Trust

To reduce measurement error and following research recommending against using only a single question to measure trust, we performed a principal component analysis of five separate trust measures.^26^

The GWP asks respondents the question: ‘Do you have confidence in each of the following, or not? How about *each of our five variables*?’ We use the following five institutions available in the GWP: financial institutions, judicial system, national government, honesty of elections and the military. Trust has been shown to be stable and to be transmitted across generations.^27^

Supplementary Tables S2 and S3 present the details of the principal component analysis. In Supplementary Table S2 we show that the first principal component has an eigenvalue >1 and explains 69% of the total variance, while the other four explain the remaining 31%, all with eigenvalues <1. Supplementary Table S3 shows the loadings of the principal components. Here, we found that the first principal component has positive loadings of about equal size on all individual trust variables. The other components have positive and negative loadings of different magnitudes on the individual trust variables.

We then keep only the first principal component, because the eigenvalue is >1, following the Kaiser–Guttman rule. This principal component becomes our measure of trust, which, by construction, has a mean of zero. Higher values correspond to higher trust in institutions. As an alternative to our trust measure we also use a measure of corruption in the government. The precise question is: ‘Is corruption widespread throughout the government in (country), or not?’. Corruption and our principal component measure of trust have a correlation of −0.57 (p<0.001).

### Control variables

Selection of control variables is informed by the related literature and data availability.^4,6,12,28^Supplementary Material S.1 has a complete and detailed list of all variables.

### Statistical analysis

In our analysis we estimate a regression model for country *i*:


(2) \begin{eqnarray*}
{{Y}_i} = \ \alpha + \ \beta \textit{Trus}{{t}_i}\ + \theta {{X}_i} + \ {{\varepsilon }_i},\end{eqnarray*}

where we capture the linear (β) effect of trust on vaccination rates or vaccination speed (Y_i_). We found that a non-linear expression of trust is non-significant. Furthermore, we control for various confounding variables in vector *X*_i_. All regressions cluster standard errors at the country level.

To address the possibility that our regression suffers from reverse causality, we present results from an instrumental variable regression. Our instrumental variable is historic exposure to influenza. The data for total influenza cases from 1960 to 2021 is taken from the GIDEON database (https://www.gideononline.com). We divide by the population to get a measure of exposure of the population. Additionally, we employ measles and malaria as alternatives to influenza for the robustness check. The underlying idea is that as influenza cases increase, people will lose faith in institutions for failing to safeguard the populace and for allowing the health system to become overwhelmed; such effects are particularly strong during pandemics (see Kittleson and Keating for Ebola).^29–31^ We used Stata SE version 18.0 (StataCorp, College Station, TX, USA) for all analyses.

## Results

**Figure 1.**
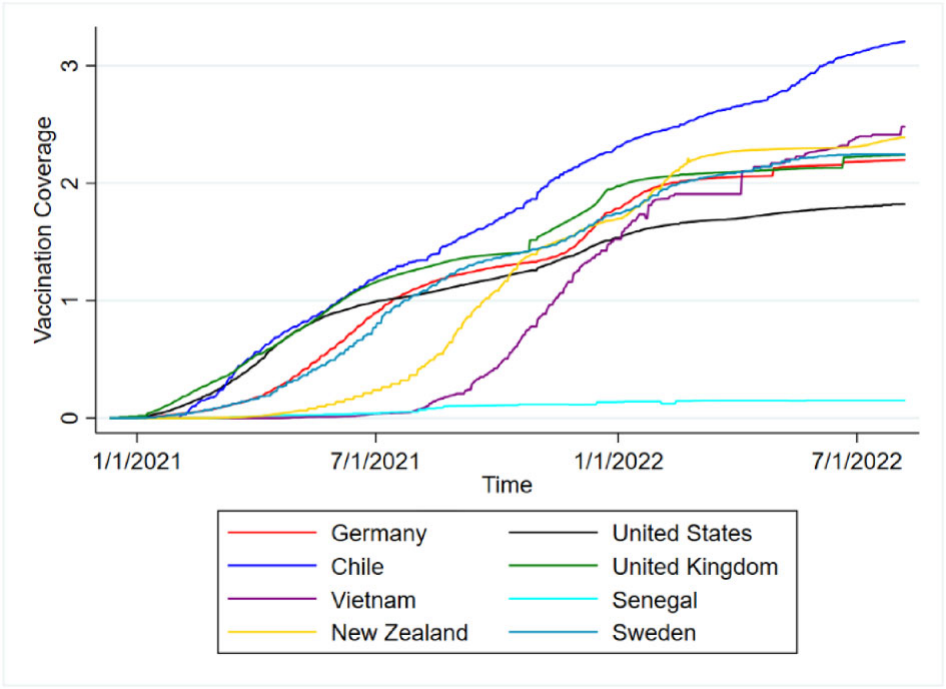
Vaccination campaigns for eight selected countries over time.

Table 1 presents the descriptive statistics of our sample and Supplementary Figure S1 shows the histogram of vaccination rates, Supplementary Figure S2 shows the histogram of vaccination speed and Supplementary Figure S3 shows the histogram for trust. In our sample, COVID-19 had an average infection rate of 15±16.1% and an average death rate (per population) of 0.1±0.1%. Note that these are officially reported cases, which almost certainly underestimates the true case numbers. The death rate in our data is similar to the global excess mortality rate, 120.3/100 000.^32^ On average, 52.8±26% of people were fully vaccinated. However, we found a large difference between the minimum of 1.5% (Yemen) and the maximum of 92.2% (Chile). The average vaccination speed was 0.007±0.005. The fastest vaccination campaign (measured in vaccines supplied) occurred in Vietnam and the slowest in Senegal. Figure 1 presents the S-shaped paths of vaccination coverage for selected countries. We selected countries with the highest and lowest vaccination speed as well some illustrative intermediate ones. Speed refers to the steepness of the S-curve.

**Table 1.** Characteristics of the countries analysed regarding the relation between trust and vaccination.

Characteristics	Obs.	Mean	Median	SD	Minimum	Maximum	Unit
Vaccination rate	114	0.528	0.591	0.260	0.015	0.922	%
Vaccination speed	104	0.007	0.006	0.005	0.001	0.022	Unitless
Trust	114	0	−0.062	1.863	−3.617	4.715	Unitless
Log (household income)	114	8.859	8.761	1.161	5.530	10.949	Log(USD)
Female	114	0.542	0.548	0.053	0.393	0.657	Dummy
Household size	114	4.314	3.929	1.799	2.110	10.782	Number
Employed	114	0.546	0.547	0.074	0.356	0.712	Dummy
Rural	114	0.270	0.229	0.180	0	0.712	Dummy
Health problems	114	0.255	0.246	0.062	0.129	0.445	Dummy
Education	114	1.810	1.807	0.327	1.148	2.447	Categorical
Religiosity	114	0.725	0.847	0.253	0.185	0.990	Dummy
Vaccine supply	114	0.890	0.949	0.488	0.043	2.206	%
Vaccine financial support	114	2.741	2.775	0.370	0.825	3.161	Categorical
Vaccine mandate	114	0.312	0	0.412	0	1	Dummy
Health expenditures	112	0.067	0.067	0.027	0.024	0.168	%
Polity2	113	5.752	8	5.028	−7	10	Number
COVID-19 deaths (share)	114	0.001	0.001	0.001	0.000	0.006	%
COVID-19 cases (share)	114	0.150	0.084	0.161	0.000	0.548	%
Gini	99	37.987	36.120	7.541	26.605	63.201	Unitless
Corruption	114	0.740	0.816	0.208	0.061	0.952	Dummy
Internet	111	0.394	0.336	0.299	0.016	0.956	Dummy
Television	111	0.814	0.962	0.258	0.153	0.996	Dummy

Obs: observations; SD: standard deviation.

Unit corresponds to the measurement unit of the variable before it was averaged to obtain the country-level average shown in this table.

Mean trust in institutions was 0±1.865. The lowest trust in institutions was found in Peru and the highest in Laos. Average vaccination supply (for two doses) was 89±48.8% and 31.2±41.2% of countries had a vaccine mandate in place. Financial support for vaccines was close to full support, i.e. the vaccination costs were fully government funded. Average (log) household income in our sample was 8.859±1.161, 54.2±5.3% were female and 54.6±7.4% were employed. Average household size was 4.314±1.799, 27±18% lived in rural areas and 25.5±6.2% of respondents had health problems. The average respondent had obtained a secondary education. On average, 72.5±25.3% of respondents state that religion is an important part of their lives. Furthermore, 74±20.8% reported corruption, 39.4±29.9% had access to the internet and 81.4±25.8% had access to a television.

**Figure 2.**
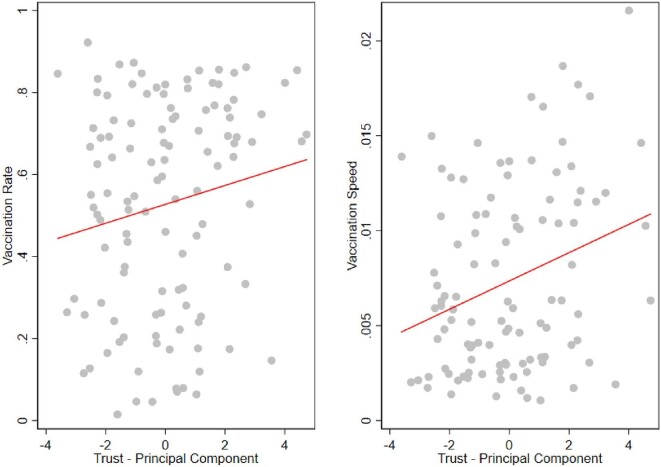
Binned scatter plot representing the relation between trust and vaccination campaign outcomes. Left panel shows the trust and vaccination rate relationship. The right panel shows the relationship of trust with vaccination speed. The red line is the linear regression line.

In Figure 2 we present two scatter plots illustrating the relationship between trust and our two vaccination campaign outcomes. The left panel depicts the association between our trust measure and the vaccination rate, while the right panel illustrates the correlation between trust and the speed of the vaccination campaign. In both figures, each data point represents a specific country. The scatter plot suggests a pattern: as trust increases, so does the vaccination rate and the speed of vaccination. To further analyse this relationship, we overlayed a red linear regression line on each plot. This line represents the unconditional correlation between trust and the respective vaccination campaign outcomes. Its upward trend reinforces the suggestive evidence we observe in the scatter plots, indicating that higher levels of trust tend to coincide with both higher vaccination rates and faster adoption of vaccination initiatives.

A different way to provide some initial idea about the relationship between trust and vaccination coverage is to compute the odds ratio (OR) of trust. We found that the OR ranges from 1.16 to 1.09 (p<0.1–<0.001) depending on which controls are included, indicating that that the odds of vaccination are 9–16% higher when people have more trust in institutions.

Finally, we also tested whether our key variables—trust, vaccination speed and coverage—are different across World Bank income groups. Using a Wald χ^2^ test on equality of means across groups allowing for heterogenous variances within groups, we found that trust (p=0.22) was not statistically significantly different across groups but that both vaccination campaign outcomes—coverage (p<0.01) and speed (p<0.01)—are significantly different across groups.

### Vaccination rates

Table 2 presents the results from our regression model (equation 2). Column 1 shows the results from a parsimonious model including only a small set of potentially important control variables, to avoid overfitting. We found that higher levels of trust in institutions correlate with higher vaccination rates (p<0.01). However, we are concerned that this regression suffers from endogeneity. As described above, we also use an instrumental variable approach. In column 2 we use an instrument (historical exposure to influenza) for the willingness to accept vaccination and other governmental public health measures. Our hypothesis that prior exposure to influenza reduces institutional trust is confirmed in the first stage. In the second stage, we found that the trust coefficient is almost the same as in column 1 and hence we believe that endogeneity plays only a minor role in this regression. These results are robust to using measles or malaria. Influenza exposure does not significantly affect COVID-19 case shares (p=0.734) or death shares (p=0.103) using a fractional logit model and core controls.

**Table 2.** Estimation results for the association between trust and vaccination rates.

Variables	(1)	(2)	(3)	(4)	(5)	(6)	(7)
Trust	0.152***	0.154***	0.364**	0.173***	0.008	0.091***	0.084**
	(0.043)	(0.052)	(0.151)	(0.056)	(0.054)	(0.026)	(0.034)
Log(household income)	0.369**	0.046	−0.519	0.674***	0.153	0.045	0.264*
	(0.161)	(0.062)	(0.376)	(0.234)	(0.258)	(0.092)	(0.139)
Female	4.827***	0.965	−0.321	5.218*	2.643	2.028**	1.034
	(1.856)	(0.704)	(5.059)	(2.657)	(2.992)	(1.026)	(1.294)
Household size	−0.298***	−0.078**	0.039	−0.284**	0.549*	−0.066	−0.050
	(0.075)	(0.033)	(0.187)	(0.125)	(0.302)	(0.044)	(0.070)
Employed	−0.964	−0.691	1.223	−0.138	−3.287*	−0.601	−0.040
	(1.070)	(0.512)	(2.276)	(1.536)	(1.720)	(0.642)	(0.900)
Rural	−1.374***	−0.592**	−0.170	−1.103*	1.530	−0.106	0.316
	(0.458)	(0.261)	(1.537)	(0.640)	(0.933)	(0.332)	(0.495)
Health problem	−2.560*	−0.592	0.732	−2.408	−5.111***	−0.657	−0.753
	(1.363)	(0.500)	(3.581)	(1.783)	(1.901)	(0.789)	(0.976)
Education	−0.856*	−0.097	2.692**	−1.832***	−0.737	−0.269	−0.533
	(0.449)	(0.160)	(1.277)	(0.559)	(0.685)	(0.271)	(0.372)
Religiosity	−0.084	0.083	0.174	−0.006	−1.927***	0.086	−0.465
	(0.435)	(0.143)	(4.875)	(0.747)	(0.529)	(0.300)	(0.354)
Vaccine supply						2.034***	1.805***
						(0.236)	(0.242)
Vaccine financial support						0.036	−0.166
						(0.197)	(0.217)
Vaccine mandate						0.146	0.154
						(0.115)	(0.116)
Health expenditure							0.173
							(2.626)
Polity2							−0.012
							(0.010)
Gini							0.022**
							(0.009)
Corruption							−0.394
							(0.340)
Internet							−0.778
							(0.735)
TV							1.080***
							(0.414)
Countries	All	All	Low income	Middle income	High income	All	All
Observations	114	114	21	57	36	114	95
*R* ^2^	0.14		0.09	0.08	0.02	0.18	0.18
First stage							
Influenza (per capita)		−0.029**					
		(0.011)					
*F*-statistic		7.17					

The table presents the coefficients from a multivariate fractional logit model (except for column 2, where we use a two-stage ordinary least squares) using the vaccination rate as a dependent variable. Columns show different specifications including an instrumental variable (IV) approach (column 2), varying the countries included (columns 3–5) and the set of control variables included (columns 6 and 7). Clustered standard errors at the country level are presented in parentheses. For the country classification, we used the 2019 World Bank income thresholds: low income (≤$995), middle income (>$996–≤$12 055) and high income (>$12 056). Significance levels: *p<0.10, **p<0.05, ***p<0.01.

Columns 3–5 run the regression for low-, middle- and high-income countries separately. We found that the impact of trust on the vaccination rate is particularly strong in low-income countries (p<0.05), while it is smaller but still important in middle-income countries (p<0.01). In contrast, we did not find a statistically significant effect in high-income countries.

Our controls are in line with the related literature. We found that higher income levels are associated with higher vaccination rates.^8,33^ We also found that household size negatively affects vaccination rates. This is a well-known finding in the vaccination literature.^34^ Countries with more population living in rural areas have lower vaccination rates. Either supply chain issues (given storage and transportation requirements) or less information about the need for vaccination in those areas may explain these results.^35^ Education has no impact in high-income countries, but it has a significantly positive effect in low-income countries (p<0.05) and a significantly negative effect in middle-income countries (p<0.01).

Columns 6 and 7 add additional control variables to our preferred model specification. We found that the trust effect becomes smaller but is still quantitatively important and statistically significant. To be precise, column 6 adds other vaccine policies from the Oxford Covid-19 Government Response Tracker. We found that only vaccine coverage (vaccine supply) has the expected positive effect on vaccination rates (p<0.01). Financial support for vaccination or vaccine mandates do not have a significant effect on vaccination rates. In column 7, higher income inequality is associated with increases in vaccination rates (p<0.05) and television access is also correlated with vaccination rates (p<0.01).

### Vaccination speed

An important factor in fighting a global pandemic like COVID-19 is not only how many people are vaccinated, but how quickly people receive the vaccine, thus lowering transmission rates. Table 3 presents the results for the speed of the vaccination campaign.

**Table 3.** Estimation results for the association between trust and vaccination speed.

Variables	(1)	(2)	(3)	(4)	(5)	(6)	(7)
Trust	0.079***	0.501***	0.186***	0.093**	0.045	0.073***	0.069**
	(0.023)	(0.183)	(0.013)	(0.040)	(0.039)	(0.017)	(0.031)
Log(household income)	0.167**	-0.014	0.051	0.267**	0.164	0.089	0.059
	(0.072)	(0.201)	(0.056)	(0.109)	(0.176)	(0.055)	(0.113)
Female	1.423*	1.739	−9.681***	1.542	3.676	0.271	0.304
	(0.788)	(2.413)	(1.313)	(1.035)	(2.883)	(0.573)	(0.771)
Household size	−0.087***	−0.148	−0.150***	−0.096**	0.434**	−0.023	0.011
	(0.030)	(0.113)	(0.037)	(0.043)	(0.199)	(0.025)	(0.036)
Employed	−0.001	−2.430	−3.982***	0.323	−2.498*	−0.350	−0.377
	(0.571)	(2.129)	(0.715)	(0.713)	(1.380)	(0.430)	(0.597)
Rural	−0.461**	−1.402	0.515***	−0.462	0.436	−0.085	0.019
	(0.202)	(0.956)	(0.161)	(0.287)	(0.692)	(0.183)	(0.305)
Health problem	−1.597**	−2.033	0.171	−1.091	−3.349**	−0.870*	−1.279**
	(0.678)	(1.481)	(0.312)	(0.882)	(1.386)	(0.483)	(0.573)
Education	−0.665**	−0.146	1.468***	−0.742*	−0.283	−0.407**	−0.320
	(0.271)	(0.600)	(0.212)	(0.384)	(0.508)	(0.201)	(0.253)
Religiosity	−0.315	−0.000	10.571***	−0.020	−1.281***	−0.244	−0.374
	(0.313)	(0.465)	(1.934)	(0.642)	(0.387)	(0.245)	(0.332)
Vaccine supply						0.752***	0.669***
						(0.127)	(0.149)
Vaccine financial support						−0.463**	−0.376*
						(0.180)	(0.209)
Vaccine mandate						0.015	−0.014
						(0.073)	(0.077)
Health expenditure							1.426
							(2.135)
Polity2							−0.010
							(0.007)
Gini							0.006
							(0.006)
Corruption							−0.103
							(0.279)
Internet							−0.092
							(0.436)
Television							0.399
							(0.331)
Countries	All	All	Low income	Middle income	High income	All	All
Observations	104	104	12	56	36	104	88
*R* ^2^	0.54		0.99	0.43	0.58	0.73	0.73
First stage							
Influenza (per capita)		−0.026**					
		(0.013)					
*F*-statistic		6.49					

The table presents the coefficients from a multivariate fractional logit model (except for column 2, where we use a two-stage ordinary least squares) using the vaccination speed*100 as a dependent variable. Columns show different specifications including an IV approach (column 2), varying the countries included (columns 3–5),and the set of control variables included (columns 6 and 7). Clustered standard errors at the country level are presented in parentheses. For the country classification, we used the 2019 World Bank income thresholds: low income (≤$995), middle income (>$996–≤$12 055) and high income (>$12 056). Significance levels: *p<0.10, **p<0.05, ***p<0.01.

Column 1 presents the results from the parsimonious model. We found that higher levels of institutional trust are associated with a faster vaccination campaign (p<0.01). When we move to our instrumental variable estimation in column 2, we found that this coefficient is underestimated and is much larger when addressing the potential endogeneity issue (p<0.01). As before, we found that trust has the largest association with the vaccination speed in low- and middle-income countries, while it has no effects in high-income countries.

When we control for other vaccination policies (column 6), we see that the trust effect is still significant and, as expected, the supply of vaccines increases the speed of the campaign (p<0.01). We also found that financial support reduces the speed of the campaign (p<0.05). We speculate that concerns about quality drive this result, given the well-known finding in experimental economics that people use prices as a signal of quality, especially when they have imperfect information.^35^ Including further controls in column 7 does not change our results.

For our controls, we found that higher household incomes are associated with faster vaccination speed. Further, household size reduces the vaccination speed, which contradicts the results of Ngo et al.^28^ A larger share of rural population reduces the vaccination speed, probably because of transportation and information challenges.^36^ Also, we found that a larger share of people with health problems reduces vaccination speed. This could be because people with comorbidities have higher vaccine hesitancy, so they take more time to observe, collect information and decide to take the vaccine.^37^ Finally, education, again, has a positive effect in low-income countries (p<0.01) but a negative effect in middle-income countries (p<0.10). The negative effect is in line with earlier research results.^28^

## Discussion

This article looked at the relationship between trust in institutions and vaccination campaign outcomes in 114 countries and documents heterogeneity in the relationship across development groups. We test whether trust in institutions predicts how many people get vaccinated and how quickly the campaign progresses. Existing research indicates a correlation between COVID-19 vaccinations and vaccination intentions; however, our research is distinct in that it leverages actual vaccination campaign outcomes from a large number of countries.

Our key findings are that higher trust in institutions significantly increases the vaccination rate and the vaccination speed in our sample, after accounting for a large number of potential confounding variables. In fact, our findings indicate that, besides trust, there are essentially no other relevant factors (other than the number of vaccines supplied, which is to be expected). This demonstrates the significance of trust to vaccination campaigns.

The effect is particularly strong in low- and middle-income countries and is not significant in high-income countries. Again, trust is one of the few elements that influences the vaccination rate. In high-income countries, health problems reduce vaccination rates, which we believe is a result of how the costs and benefits of vaccines are communicated to the population. Higher education increases vaccination rates in low-income nations, which is again related to how the costs and benefits of vaccines are communicated. In middle-income countries, vaccination rates are negatively correlated with household size, rurality and education. This underscores the necessity of distributing vaccines as well as how to communicate the vaccination's benefits with varied audiences (e.g. by educational attainment).

For the speed of the vaccination campaign, we found that the factors that influence it vary considerably more between development groups. In high-income countries we again found that health problems increase hesitance (reduce vaccination speed). In middle-income countries, income increases vaccination speed, but household size reduces speed. In low-income countries, the relationship between vaccination speed and rurality, education and religiosity are positive, whereas it is negative for gender, household size and employment. Overall, uptake of the vaccine is determined by a greater number of factors than the overall vaccination rate. However, trust is always a significant factor.

### Limitations

Several limitations of our study need to be considered. First, we lack data on trust in other relevant factors, such as medical professionals or science in general. This could lead to an overestimation of the effect of trust in institutions. However, we believe there will be a strong correlation between these factors with institutional trust. Second, individual-level vaccination data would clearly be superior to country-level data and would reduce measurement error. Finally, with high-frequency data on vaccinations the analysis could be conducted differently by estimating panel methods. Such data could complement our approach to estimating the speed of the vaccination campaign. Future research should build on our results by addressing these limitations.

### Public health implications

Our findings have implications for the design of vaccination campaigns for national governments and international organizations. The findings highlight the importance of trust in institutions when designing communication strategies around vaccination campaigns.^38^ Our results suggest that developing country-specific communication strategies that promote trust can increase vaccine uptake, especially in low- and middle-income countries. Furthermore, our findings suggest the need for further research into the rate of vaccine adoption, beyond the obvious issue of supply, particularly for viruses with a rapid mutation rate, such as SARS-CoV-2.

## Supplementary Material

trae048_Supplemental_File

## Data Availability

We cannot make the Gallup data available. The data were obtained following a successful application to a competitive process that awarded a Gallup World Poll data license and access to microdata files. The data access agreement stipulates that the microdata cannot be redisseminated by users or shared with anyone other than the individuals who are granted access to the microdata.
