# Supplementary material for: Trust in institutions affects vaccination campaign outcomes
Source: Trans R Soc Trop Med Hyg. 2024 Jul 30;118(11):720–8. doi: 10.1093/trstmh/trae048 (PMC11532736; doi:10.1093/trstmh/trae048)
Supplement: trae048_Supplemental_File [file trae048_supplemental_file.docx]

**Supplementary Material**

*S.1 Variable Description*

Our first control variable is household income. Household income is initially reported in the respondent’s local currency and then transformed into international dollars using the Bank’s PPP (2011) private consumption conversion factor. Further, we use the country-level Gini index as a measure of the inequality in the distribution of household income. Employment status is a dummy variable, which equals one if the respondent is employed (self-employed, full-time or part-time) and zero, if the respondent is unemployed or out of the workforce.

Location can affect infection risk and access to health care as well as vaccinations [35]. Therefore, we include a rural dummy, which is one if the respondent lives in a rural area and zero if they live in a small town or village or large city or a suburb. [28] show that education can determine the speed of vaccinations. Our education variable takes on three values: one for elementary education, two for secondary education, and three for postsecondary education. Research has shown COVID-19 mortality varies substantially by sex. Therefore, we include an indicator for whether the respondent is female.^[[1]](#footnote-1)^ Further, research has shown that comorbidities are a risk factor for severe COVID-19 cases and deaths. We include the prevalence of health problems derived from responses to the question “*Do you have any health problems that prevent you from doing any of the things people your age normally can do?*”. The number of people in a household (adults and children) is an additional control, as it relates to the risk of infection. Further, religiosity can affect the perceptions about vaccines. We therefore include a religiosity variable based on the survey question: “*Is religion an important part of your daily life?*”. Media sources can affect vaccine hesitancy. We therefore include two variables measuring access to internet and having a television at home.

We also include a measure of political freedom: the polity2 variable created by the *Center for Systemic Peace* under the Polity™ IV project. This variable captures political freedom and varies between -10, indicating a strong autocracy, and 10, indicating a strong democracy. We use the latest available data for 2018 for each country.

The total number of doses of all brands of vaccines delivered to each country in our sample is taken from the UNICEF COVID-19 Vaccine Market Dashboard. We divide this variable by the population times two (because in most cases it takes two doses to get full protection) to get the vaccine supply per capita (the results are robust to dividing by the population times three or four and not adjusting for the population). Two additional vaccine supply measures were taken from the data on vaccine policies from the Blavatnik School of Government at the University of Oxford (The Oxford Covid-19 Government Response Tracker, OxCGRT for short) for January 1, 2020 to August 5, 2022, following [28]. First, we use a measure of the national average financial support for vaccinations over time. Financial support can have three values: no support (individuals pay full cost), partial public funding, total public funding. Second, we construct a variable indicating the usage of vaccine mandates for each country over time. The underlying variable is a dummy variable indicating whether a mandate is in place. We then take the average for each country over time. A larger value of this variable indicates that a government vaccination mandate has been in place for a longer period.

*S.2 Additional Figures*

Figure S1: Histogram for Vaccination Rate

**
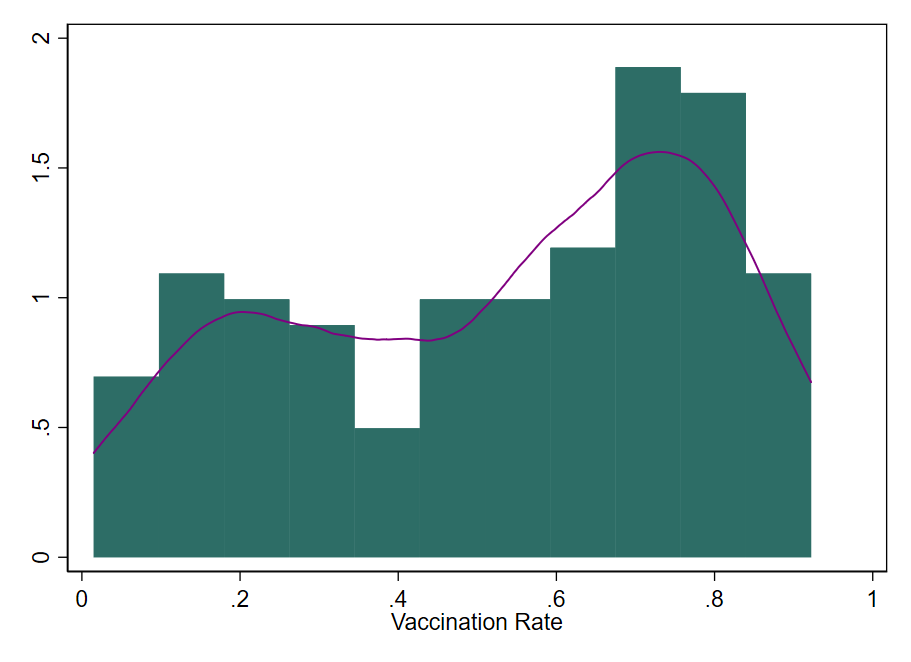
**

Figure S2: Histogram for Vaccination Speed

**
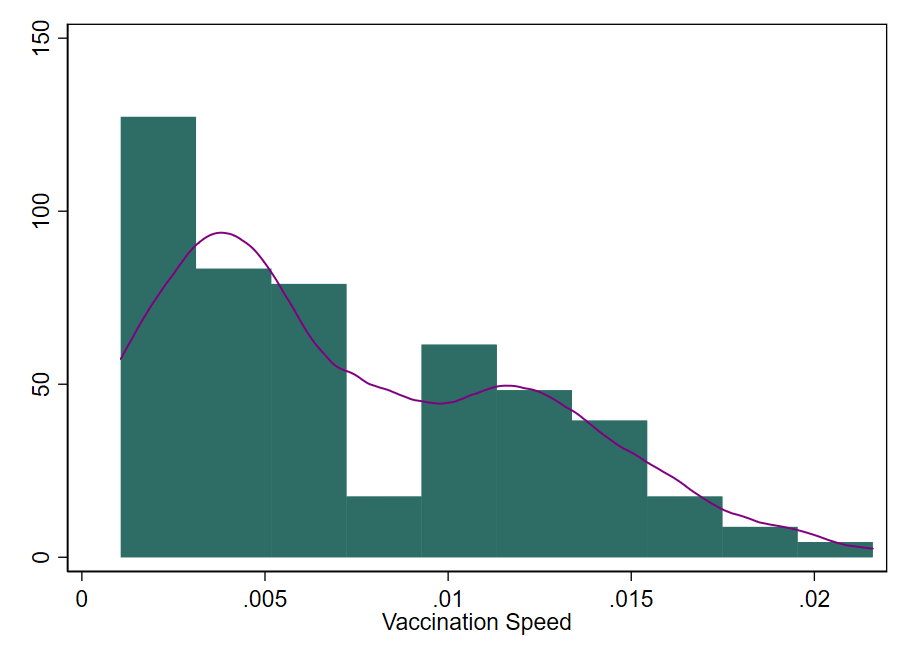
**

Figure S3: Histogram for Trust

**
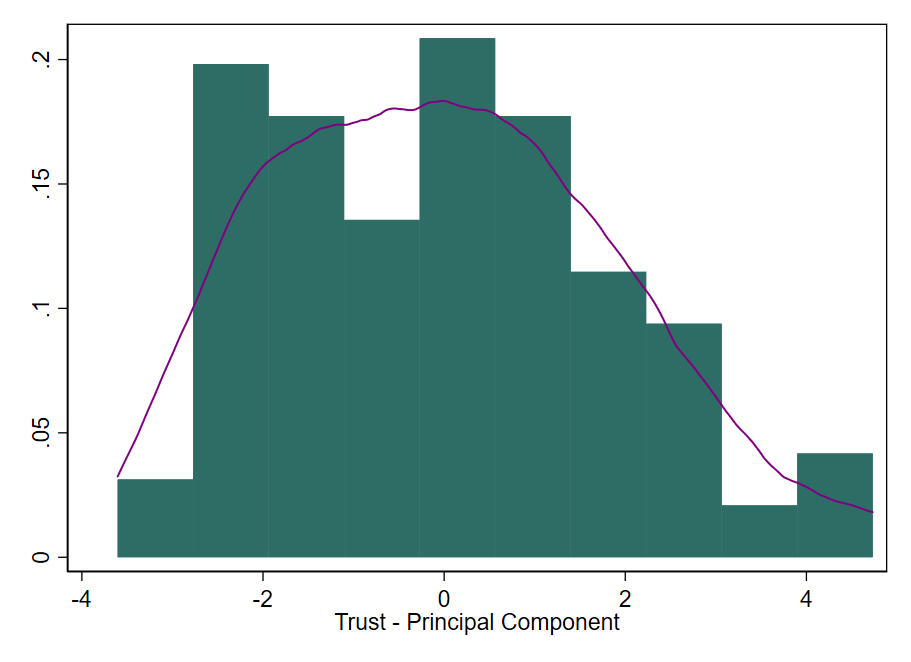
**

Table S1: List of Countries

| Afghanistan | Chile | Ghana | Latvia | Niger | Spain |
| --- | --- | --- | --- | --- | --- |
| Albania | Colombia | Greece | Lesotho | Nigeria | Sri Lanka |
| Argentina | Costa Rica | Guatemala | Liberia | Norway | Sweden |
| Australia | Cote d’Ivoire | Guinea | Lithuania | Pakistan | Switzerland |
| Austria | Croatia | Honduras | Luxembourg | Panama | Tajikistan |
| Azerbaijan | Cyprus | Hungary | Madagascar | Paraguay | Tanzania |
| Belarus | Denmark | India | Malawi | Peru | Thailand |
| Belgium | Dominican Republic | Indonesia | Malaysia | Philippines | Togo |
| Benin | Ecuador | Iran | Mali | Poland | Tunisia |
| Bolivia | Egypt | Iraq | Mauritania | Portugal | Turkey |
| Bosnia and Herzegovina | El Salvador | Ireland | Mauritius | Romania | Uganda |
| Botswana | Estonia | Israel | Mexico | Russia | UK |
| Brazil | Ethiopia | Italy | Moldova | Rwanda | USA |
| Bulgaria | Finland | Jamaica | Mongolia | Senegal | Uruguay |
| Burkina Faso | France | Japan | Mozambique | Serbia | Venezuela |
| Cambodia | Gabon | Kazakhstan | Namibia | Sierra Leone | Vietnam |
| Cameroon | Gambia | Kenya | Netherlands | Singapore | Yemen |
| Canada | Georgia | Kyrgyzstan | New Zealand | Slovakia | Zambia |
| Chad | Germany | Laos | Nicaragua | South Africa | Zimbabwe |

Table S2: Principal Component Analysis I (Eigenvalues)

| Component | Eigenvalue | Proportion | Cumulative |
| --- | --- | --- | --- |
| 1 | 3.470 | 0.694 | 0.694 |
| 2 | 0.749 | 0.150 | 0.844 |
| 3 | 0.447 | 0.089 | 0.933 |
| 4 | 0.195 | 0.039 | 0.972 |
| 5 | 0.139 | 0.028 | 1.000 |

Table S3: Principal Component Analysis II (Factor Loadings)

| Trust in: | Component 1 | Component 2 | Component 3 | Component 4 | Component 5 |
| --- | --- | --- | --- | --- | --- |
| Military | 0.404 | -0.511 | 0.715 | -0.197 | 0.162 |
| Judicial System | 0.500 | -0.147 | -0.101 | 0.448 | -0.720 |
| Government | 0.474 | 0.331 | -0.200 | -0.768 | -0.188 |
| Financial Institutions | 0.397 | 0.700 | 0.308 | 0.386 | 0.331 |
| Elections | 0.452 | -0.344 | -0.587 | 0.148 | 0.558 |

1. The data does not include nonbinary as an option. [↑](#footnote-ref-1)
